# Forest Bathing in Urban Green Spaces: Impact on Wellbeing of Family Caregivers of People Living with Dementia

**DOI:** 10.1093/geroni/igaf122.673

**Published:** 2025-12-31

**Authors:** Jacky C P Choy, Maggie S L Ma

**Affiliations:** The University of Hong Kong, Hong Kong, China (People’s Republic); The University of Hong Kong, Hong Kong, China (People’s Republic)

## Abstract

Forest bathing is an emerging approach aimed at addressing anxiety, depression, and overall psychological wellbeing. Given the needs of family caregivers of people living with dementia (PLwD) and the availability of public green spaces in Hong Kong, we adapted the forest bathing intervention for family caregivers and implemented it in parks in the urban areas. This study aims to assess its acceptability, feasibility, and effectiveness in reducing caregivers’ burden, depressive symptoms, and anxiety symptoms. We conducted a non-randomized controlled trial with 107 dyads of family caregivers and PLwD in the community. The experimental group (n = 54) participated in three sessions of the forest bathing intervention, while the control group (n = 53) received the same number of sessions of art activity intervention. Data on attrition, satisfaction, adherence, and changes in primary outcomes over six weeks were collected and analysed. The intervention protocol was highly acceptable and feasible, with a 17% attrition rate, 89% of participants perceiving benefits for their wellbeing, 75% adhering to the homework protocol, and accessible green spaces in the neighbourhood at no additional cost. Compared to the control group, caregivers who participated in the forest bathing intervention reported lower levels of care burden, depressive symptoms, and anxiety symptoms, after controlling for demographic factors. The findings support the forest bathing intervention as an evidence-based group activity in community caregiver support services. Further investigations are needed to explore its mechanisms of change, particularly the dyadic participation of caregivers and PLwD, as well as the homework component that encourages self-practice.

